# Supplementary material for: A fault-intrusion-tolerant system and deadline-aware algorithm for scheduling scientific workflow in the cloud
Source: PeerJ Comput Sci. 2021 Nov 2;7:e747. doi: 10.7717/peerj-cs.747 (PMC8576554; doi:10.7717/peerj-cs.747)
Supplement: Supplemental Information 6 [file peerj-cs-07-747-s006.zip › Myproject1.1/docs/org/cloudbus/cloudsim/provisioners/BwProvisionerSimple.html]

BwProvisionerSimple


JavaScript is disabled on your browser.


- Overview
- Package
- Class
- Use
- Tree
- Deprecated
- Index
- Help

- Prev Class
- Next Class

- Frames
- No Frames

- All Classes

- Summary:
- Nested |
- Field |
- Constr |
- Method

- Detail:
- Field |
- Constr |
- Method


org.cloudbus.cloudsim.provisioners

## Class BwProvisionerSimple

- java.lang.Object
- - org.cloudbus.cloudsim.provisioners.BwProvisioner
  - - org.cloudbus.cloudsim.provisioners.BwProvisionerSimple

- ---

    

  ```
  public class BwProvisionerSimple
  extends BwProvisioner
  ```

  BwProvisionerSimple is a class that implements a simple best effort allocation policy: if there
  is bw available to request, it allocates; otherwise, it fails.

  Since:
  :   CloudSim Toolkit 1.0

- - ### Constructor Summary

    Constructors

    Constructor and Description

    `BwProvisionerSimple(long bw)`

    Instantiates a new bw provisioner simple.
  - ### Method Summary

    Methods

    | Modifier and Type | Method and Description |
    | --- | --- |
    | `boolean` | `allocateBwForVm(Vm vm, long bw)` Allocates BW for a given VM. |
    | `void` | `deallocateBwForAllVms()` Releases BW used by a all VMs. |
    | `void` | `deallocateBwForVm(Vm vm)` Releases BW used by a VM. |
    | `long` | `getAllocatedBwForVm(Vm vm)` Gets the allocated BW for VM. |
    | `protected java.util.Map<java.lang.String,java.lang.Long>` | `getBwTable()` Gets the bw table. |
    | `boolean` | `isSuitableForVm(Vm vm, long bw)` Checks if BW is suitable for vm. |
    | `protected void` | `setBwTable(java.util.Map<java.lang.String,java.lang.Long> bwTable)` Sets the bw table. |

    - ### Methods inherited from class org.cloudbus.cloudsim.provisioners.BwProvisioner

      `getAvailableBw, getBw, getUsedBw, setAvailableBw, setBw`
    - ### Methods inherited from class java.lang.Object

      `clone, equals, finalize, getClass, hashCode, notify, notifyAll, toString, wait, wait, wait`

- - ### Constructor Detail


    - #### BwProvisionerSimple

      ```
      public BwProvisionerSimple(long bw)
      ```

      Instantiates a new bw provisioner simple.

      Parameters:
      :   `bw` - the bw
  - ### Method Detail


    - #### allocateBwForVm

      ```
      public boolean allocateBwForVm(Vm vm,
                            long bw)
      ```

      **Description copied from class: `BwProvisioner`**

      Allocates BW for a given VM.

      **Specified by:**
      :   `allocateBwForVm` in class `BwProvisioner`

      Parameters:
      :   `vm` - virtual machine for which the bw are being allocated
      :   `bw` - the bw

      Returns:
      :   $true if the bw could be allocated; $false otherwise


    - #### getAllocatedBwForVm

      ```
      public long getAllocatedBwForVm(Vm vm)
      ```

      **Description copied from class: `BwProvisioner`**

      Gets the allocated BW for VM.

      **Specified by:**
      :   `getAllocatedBwForVm` in class `BwProvisioner`

      Parameters:
      :   `vm` - the VM

      Returns:
      :   the allocated BW for vm


    - #### deallocateBwForVm

      ```
      public void deallocateBwForVm(Vm vm)
      ```

      **Description copied from class: `BwProvisioner`**

      Releases BW used by a VM.

      **Specified by:**
      :   `deallocateBwForVm` in class `BwProvisioner`

      Parameters:
      :   `vm` - the vm


    - #### deallocateBwForAllVms

      ```
      public void deallocateBwForAllVms()
      ```

      **Description copied from class: `BwProvisioner`**

      Releases BW used by a all VMs.

      **Overrides:**
      :   `deallocateBwForAllVms` in class `BwProvisioner`


    - #### isSuitableForVm

      ```
      public boolean isSuitableForVm(Vm vm,
                            long bw)
      ```

      **Description copied from class: `BwProvisioner`**

      Checks if BW is suitable for vm.

      **Specified by:**
      :   `isSuitableForVm` in class `BwProvisioner`

      Parameters:
      :   `vm` - the vm
      :   `bw` - the bw

      Returns:
      :   true, if BW is suitable for vm


    - #### getBwTable

      ```
      protected java.util.Map<java.lang.String,java.lang.Long> getBwTable()
      ```

      Gets the bw table.

      Returns:
      :   the bw table


    - #### setBwTable

      ```
      protected void setBwTable(java.util.Map<java.lang.String,java.lang.Long> bwTable)
      ```

      Sets the bw table.

      Parameters:
      :   `bwTable` - the bw table


- Overview
- Package
- Class
- Use
- Tree
- Deprecated
- Index
- Help

- Prev Class
- Next Class

- Frames
- No Frames

- All Classes

- Summary:
- Nested |
- Field |
- Constr |
- Method

- Detail:
- Field |
- Constr |
- Method
